# Genomic Diversity and Evolution of SARS-CoV-2 Lineages in Pakistan

**DOI:** 10.3390/v15071450

**Published:** 2023-06-27

**Authors:** Muhammad Waqar Aziz, Nadia Mukhtar, Aftab Ahamd Anjum, Muhammad Hassan Mushtaq, Muhammad Adnan Ashraf, Amar Nasir, Muhammad Furqan Shahid, Muhammad Nawaz, Muhammad Zubair Shabbir, Noreen Sarwar, Rabia Tanvir, Tahir Yaqub

**Affiliations:** 1 Institute of Microbiology, University of Veterinary and Animal Sciences, Lahore 54000, Pakistan; waqar.aziz@uvas.edu.pk (M.W.A.); nadia.mukhtar@uvas.edu.pk (N.M.); aftab.anjum@uvas.edu.pk (A.A.A.); adnan.ashraf@uvas.edu.pk (M.A.A.); furqan.shahid@uvas.edu.pk (M.F.S.); muhammad.nawaz@uvas.edu.pk (M.N.); shabbirmz@uvas.edu.pk (M.Z.S.); noreen.sarwar@uvas.edu.pk (N.S.); rabia.tanvir@uvas.edu.pk (R.T.); 2 Department of Epidemiology, University of Veterinary and Animal Sciences, Lahore 54000, Pakistan; hassan.mushtaq@uvas.edu.pk; 3 Department of Clinical Sciences, Sub Campus Jhang, University of Veterinary and Animal Sciences, Lahore 54000, Pakistan; amar.nasir@uvas.edu.pk; 4 Veterinary Research Institute, Lahore 53810, Pakistan

**Keywords:** genomic diversity, evolution, SARS-CoV-2, lineages, variants, phylogenetic

## Abstract

The emergence of SARS-CoV-2 variants has posed a challenge to disease control efforts worldwide. This study explored the genomic diversity and phylogenetic relationship of SARS-CoV-2 variants reported in Pakistan. Our objective was to understand the transmission dynamics of different lineages within the country. We retrieved and analyzed spike protein sequences from Pakistan and compared them with reference sequences reported worldwide. Our analysis revealed the clustering of Pakistan-origin isolates in nine different clades representing different regions worldwide, suggesting the transmission of multiple lineages within the country. We found 96 PANGO lineages of SARS-CoV-2 in Pakistan, and 64 of these corresponded to 4 WHO-designated variants: Alpha, Beta, Delta, and Omicron. The most dominant variants in Pakistan were Alpha (B.1.1.7), Beta (B.1.351), Delta (B.1.617.2, AY.108), and Omicron (BA.2.75, BA.5.2), and the N-terminal domain and receptor binding regions were the most hypervariable regions of the spike gene. Compared to the reference strain, characteristic substitutions were found in dominant variants. Our findings emphasize the importance of continuously monitoring and assessing nucleotide and residue substitutions over time to understand virus evolutionary trends better and devise effective disease control interventions.

## 1. Introduction

SARS-CoV-2, a novel coronavirus, was first identified in 2019 and rapidly became a global public health concern. It was initially reported in Pakistan in February 2020, and by mid-2020, the disease had spread nationwide, with clinical cases being observed throughout the country. SARS-CoV-2 had infected 1,577,213 individuals in Pakistan, resulting in 30,643 deaths [source: https://covid.gov.pk/ (accessed on 7 March 2023)].

The spike protein is a critical structural component of SARS-CoV-2 that facilitates viral entry into host cells. The spike protein comprises 1273 amino acids and 2 primary subdomains, S1 and S2. The S1 subdomain contains the receptor-binding domain (RBD), which mediates the attachment of the virus to the ACE2 receptor on host cells. In contrast, the S2 subdomain facilitates the fusion of the virus and the host cell membrane, enabling the virus to enter the cell efficiently [1]. The S1 subunit of the spike protein is less conserved than S2 due to the high frequency of interactions. The N-terminal domain (NTD) and the receptor-binding domain (RBD) are two highly immunogenic regions on the S1 subunit, and they are the primary targets of neutralizing monoclonal and polyclonal antibodies. The ongoing spread of SARS-CoV-2 in immune-competent populations has led to novel virus variants that have evolved to adapt to the host.

The spike protein plays a crucial role in determining the outcomes of COVID-19 vaccines in terms of success and failure. As a result, the World Health Organization (WHO) classifies SARS-CoV-2 variants based on the frequency and location of substitutions in the spike protein.

COVID-19 has spread through Pakistan in five waves to date. The first wave, which emerged with the report of the Alpha variant in late May 2020, peaked in the middle of June with the highest number of newly confirmed cases and daily mortality and rapidly ended in the middle of July. The second wave began in early November 2020 with the emergence of the Beta and Mu variants. The third wave, with the emergence of the highly transmissible Delta variant, was reported in the middle of March 2021. Pakistan then encountered two additional waves: the fourth from July to September 2021 and the fifth from January to March 2022 [2]. The Delta and Omicron variants were the dominant SARS-CoV-2 variants during Pakistan’s fourth and fifth COVID-19 waves.

The occurrence of multiple variants during COVID-19 waves has led to varying consequences regarding disease occurrence over time. This may be attributed to the emergence of escape mutants or vaccine failure in vaccinated individuals or those previously infected, resulting in varying variants with varying rates of disease occurrence. The lack of data on the genetic and antigenic characteristics of prevailing variants/lineages and their spatial distribution in susceptible populations has made developing and implementing effective disease control interventions challenging. Therefore, it is crucial to understand the ongoing genetic evolution in prevailing variants, coupled with comparative residue analysis, including reference vaccine strains used in the country. With this background, we explored the COVID-19 sequence database to identify circulating lineages and clades and assess the ongoing evolution among SARS-CoV-2 in Pakistan. We used spike gene sequence data for phylogenomic clustering, elucidation of substitutions, and evolutionary trends among dominant lineages/variants circulating in Pakistan and reference prototypes reported worldwide.

## 2. Methodology

### 2.1. Sequence Dataset

A total of 1496 nucleotide sequences of the spike protein of SARS-CoV-2 from Pakistan were obtained from the NCBI database [https://www.ncbi.nlm.nih.gov/sars-cov-2/ (accessed on 5 March 2023)], see Table S1. The sequences were initially grouped into 96 lineages and 1 unclassified group according to the Phylogenetic Assignment of Named Global Outbreak Lineages (PANGOLIN) nomenclature. The complete spike gene sequences were retrieved and evaluated for sequence quality, and 38 lineages with ambiguous bases (>1% of NNNs) were excluded from phylogeny and comparative genome analysis to minimize the risk of false positive results. The final dataset for this study included SARS-CoV-2 strains representing 58 lineages. The hCoV-2/Wuhan/WIV04/2019 (NC_045512.2) was used as a reference sequence and retrieved from the NCBI database [https://www.ncbi.nlm.nih.gov/sars-cov-2/ (accessed on 5 March 2023)]. The sequences were aligned using the ClustalW multiple alignment method implemented in BioEdit^®^ version 7.2 software [3].

### 2.2. Bioinformatics Analysis

For the phylogenetic analysis, 58 complete spike protein sequences representing different circulating lineages in Pakistan and 158 from other geographical regions were used as input. A spike-protein-based phylogenetic tree was constructed using the FastTree algorithm in Geneious v7.1.9 (Biomatters Ltd., Auckland, New Zealand).

Comparative residue analysis of the spike protein was also performed using 58 complete spike protein sequences representing 58 different lineages to evaluate the presumptive role of substitutions. The open reading frame (ORF) was predicted by translating the nucleotide sequences into amino acid sequences for the entire coding region of the spike protein using BioEdit^®^ software. The amino acid sequences were aligned using the ClustalW multiple alignment method implemented in BioEdit^®^. All substitutions in the spike protein were identified manually on BioEdit^®^ and using the GISAID CoVsurver mutations application [https://gisaid.org/database-features/covsurver-mutations-app/ (accessed on 15 March 2023)] and mapped onto the spike protein gene structure. The spike protein gene structure was constructed and edited using Adobe Illustrator^®^ software (version 27.1).

## 3. Results

### 3.1. SARS-CoV-2 Spike Protein Sequences

**Figure 1:**
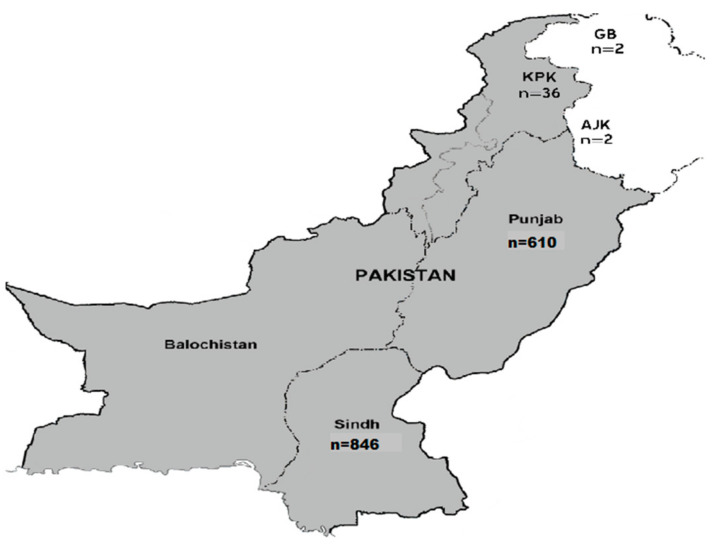
The province-wide distribution of SARS-CoV-2 sequences taken from the NCBI SARS-CoV-2 database (https://www.ncbi.nlm.nih.gov/sars-cov-2/, accessed on 5 March 2023) of Pakistan for a period spanning from March 2020 to March 2023. The map was edited by using Adobe Illustrator software (version 27.1).

Spike protein gene sequences of SARS-CoV-2 retrieved from the NCBI SARS-CoV-2 database (https://www.ncbi.nlm.nih.gov/sars-cov-2/, accessed on 5 March 2023) were reported from different geographic regions of Pakistan, and the province-wide distribution is presented in Figure 1.

**Figure 2:**
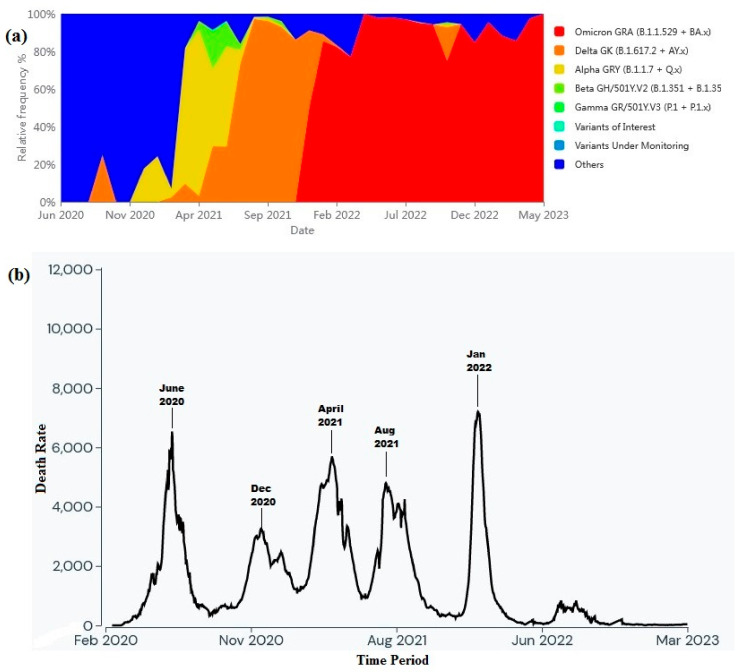
(**a**) Graph showing the temporal dynamics of variant abundance (source: https://gisaid.org/hcov-19-variants-dashboard/, accessed on 5 March 2023) and (**b**) the corresponding trend of mortality rates over time in Pakistan (source: https://outbreak.info/location-reports?loc=PAK, accessed on 5 March 2023).

It is observed that maximum SARS-CoV-2 sequences were uploaded from Sindh (*n* = 846) followed by Punjab (*n* = 610), KPK (*n* = 36), AJK (*n* = 2), and Gilgit-Baltistan (*n* = 2) provinces of Pakistan (Figure 1). The present study also provides a comprehensive analysis of the temporal dynamics between COVID-19 variants and mortality rate in Pakistan. It explores the evolving landscape of variant prevalence, emphasizing the diverse genetic strains observed within the country. Moreover, it investigates the corresponding mortality rate, offering valuable insights into the severity as shown in Figure 2.

### 3.2. Lineage Categorization of SARS-CoV-2 Genome Sequences

A total of 96 PANGO-designated lineages were circulating in Pakistan in the last three years (March 2020-March 2023), as shown in Table 1, Table 2 and Table 3. A total of 64/96 prevalent lineages correspond to 4 WHO-designated variants, including Alpha, Beta, Delta, and Omicron, as shown in Table 1 and Table 2. GISAID clades corresponding to these circulating lineages, according to the available sequence data set of SARS-CoV-2 in Pakistan, are also shown in Table 1, Table 2 and Table 3.

### 3.3. GISAID Clades Distribution of SARS-CoV-2 Sequences of Pakistan

**Figure 3:**
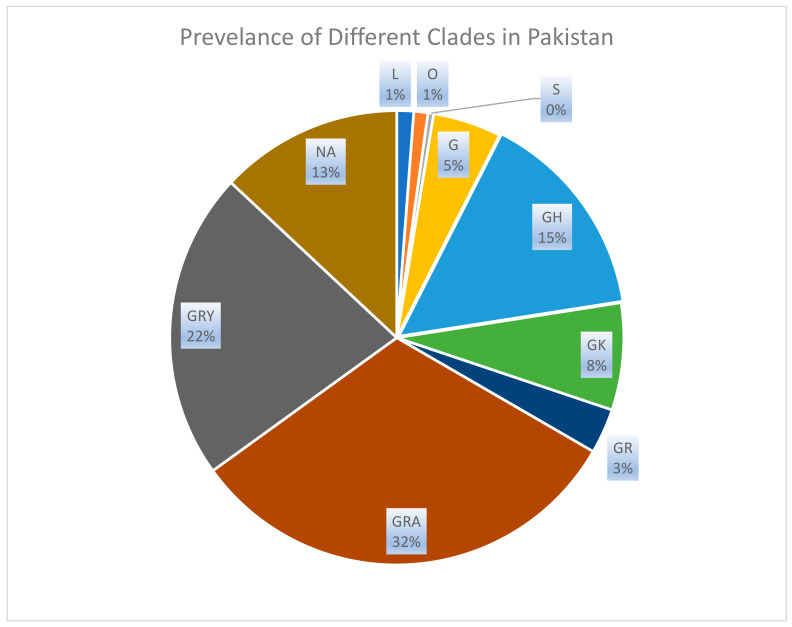
The chart represents the percentage distribution of GISAID clades observed among the SARS-CoV-2 sequences obtained from Pakistan. The names assigned to these clades—S, L, G, O, GH, GR, GRA, GK, and GRY—were designated by GISAID and are based on mutational markers observed within each clade. The data used for this analysis were downloaded on 5 March 2023. Each clade is represented by a distinct color in the chart, highlighting its relative prevalence within the sampled sequences from Pakistan.

The S protein gene sequences of SARS-CoV-2 from Pakistan were clustered in nine clades, and some sequences were found unclassifiable and not included in any clade so far. The distribution of these nine clades shows the maximum prevalence of GRA (34.27%) as shown in Table 1, followed by GRY (23.71%), GH (16.26%), GK (8.24%), G (5.27%), GR (3.47%), L (1.3%), and O (1.08%) S (0.43%) as shown in Table 2. A total of 14.09% of the sequences were under monitoring and did not represent any clade as shown in Figure 3 and Table 3.

### 3.4. Phylogenetic Analysis

**Figure 4:**
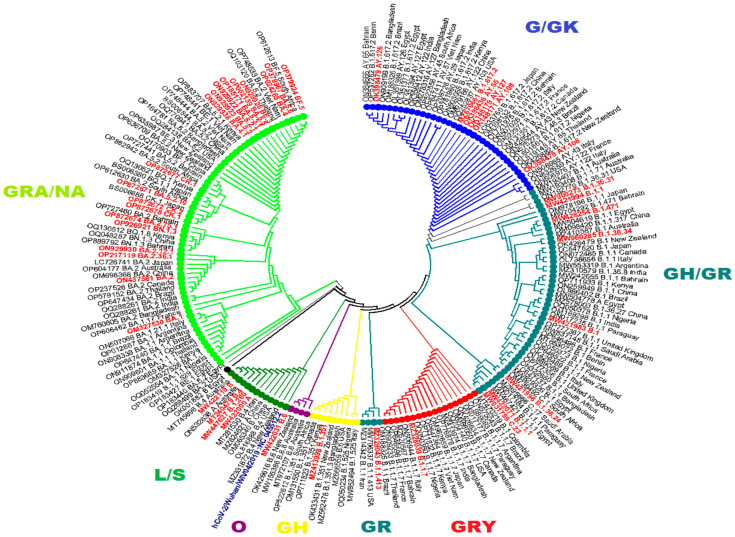
Maximum likelihood phylogenetic tree that displays the genetic relationships among sequences of SARS-CoV-2 originating from Pakistan. The sequences from Pakistan are highlighted in red. The tree shows 9 distinct clades, 1 unclassifiable group denoted as “NA”, and 58 PANGO lineages. The global SARS-CoV-2 genomes with the highest number of mutations are grouped into different clades. The phylogenetic tree also includes light green, dark green, black, purple, red, yellow, dark teal, and blue balls, which indicate the distribution of Pakistani SARS-CoV-2 samples within their respective clades.

The maximum likelihood phylogenetic tree of 58 PANGOLIN lineage S protein gene sequences of Pakistan was developed as per the classification of the GISAID clades and PANGOLIN lineage (Figure 3). The overall clades and lineages distribution represented the dominant occurrence of L (B and B.4), O (B.1.260, B.6, and B.6.6), S (A), G (B.1.617.2), GH (B.1, B.1.351, B.1.36, B.1.36.24, B.1.36.31, B.1.36.34, B.1.471, and B.1.525), GK (AY.106, AY.108, AY.126, AY.127, AY.46, AY.46.2, AY.55, and AY.65), GR (AE.4, B.1.1, B.1.1.1, B.1.1.413, and C.23), GRA (B.1.1.529, BA.1, BA.1.1, BA.1.1.1, BA.1.1.18, BA.1.15.1, BA.1.17.2, BA.1.18, BA.1.21, BA.2, BA.2.12.1, BA.2.14, BA.2.15, BA.2.3, BA.2.36.1, BA.2.5, BA.2.75.1, BA.4, BA.4.1, BA.5.2, BA.5.2.1, BA.5.2.16, BA.5.2.20, BA.5.2.28, BA.5.2.3, BA.5.2.9, BA.5.3, BA.5.3.1, BA.5.5, BA.5.6, BQ.1, BQ.1.5, BQ.1.1, BQ.1.13, XBB.1, XBB.1.11.1, XBB.1.12, XBB.1.5, XBB.1.5.9, XBB.1.9, XBB.1.9.1, XBB.1.9.2, XBB.2, XBB.2.1, and XBB.2.4), and GRY (B.1.1.7, B.1.1.7, and Q.4) clades, respectively. The maximum likelihood phylogeny tree of Pakistani and global sequence samples is presented in Figure 4.

### 3.5. Mutational Diversity in Spike Protein of SARS-CoV-2

The mutational analysis reveals that D614G amino acid change was found in high frequency and 79.5% of lineages of Pakistan. The other high-frequency substitutions present in >10% of the lineage samples include T478K (59.09%); G142D (56.81%); N501Y (50%); P681H (50%); S477N and E484A (47.72%); V211G, G339D, K417N, N440K, C498R, Y505H, H655Y, N679K, D796Y, C954H, and N969K (45.45%); S373P and S375F (44.45%); T19I, L24S, T376A, and D405N (40.90%); S371F, R408S, and F486V (29.54%); V68I (27.27%); N764K (20.45%); D950N (13.63%); and T19R, E156G, N460K, C493R, and P681R (11.36%). The rare substitutions in sub variants include R346K, K444R, L452C, E484K, N658S, A570T, A570D, T572I, D574V, N658Y, S673T, A701V, S704L, T716I, K814R, A845S, A846V, I850L, D950H, S982A, A1020S, T1117I, D1118H, E1182C, and E120C (2.27%) shown in the Table 4.

### 3.6. Comparison of Mutational Substitutions in Spike Protein in Dominant Lineages of SARS-CoV-2 in Pakistan

**Figure 5:**
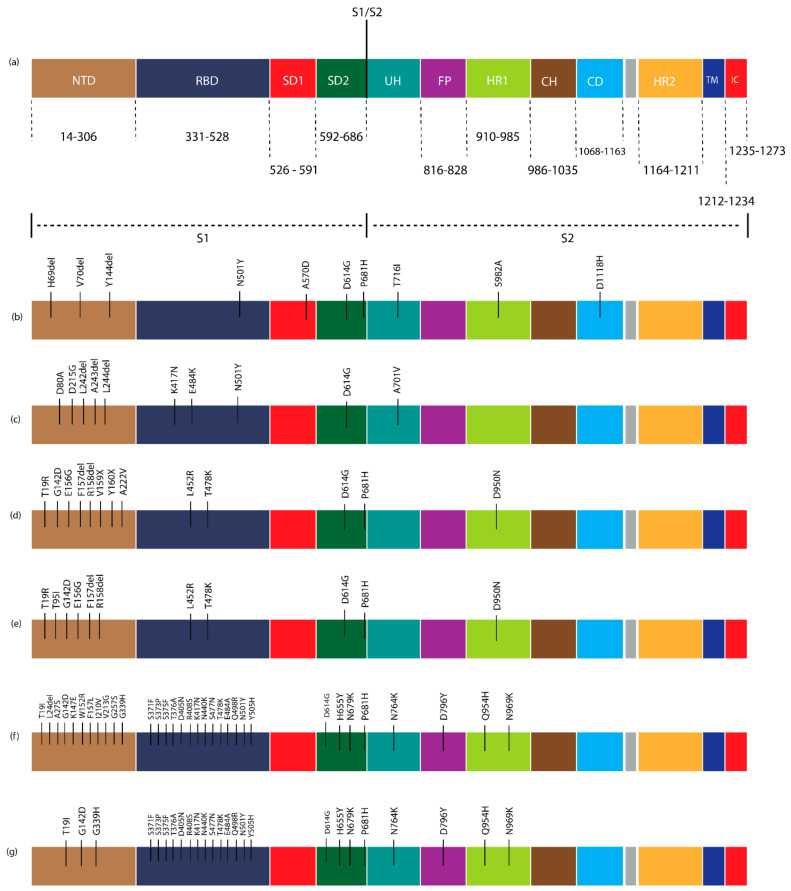
Pictorial representation of SARS-CoV-2 spike protein gene and substitutions found in dominant lineages/variants in Pakistan. The figure was constructed in Adobe Illustrator. (**a**) SARS-CoV-2 spike protein gene structure, (**b**) substitutions found in B.1.1.7/Alpha’s different regions, (**c**) B.1.351/Beta. (**d**) Substitutions found in the other regions of B.1.617.2/Delta. (**e**) Substitutions found in the different areas of AY.108/Delta and (**f**) in the different regions of BA.2.75/Omicron. (**g**) Substitutions found in the other regions of BA.5.2/Omicron.

**Figure 6:**
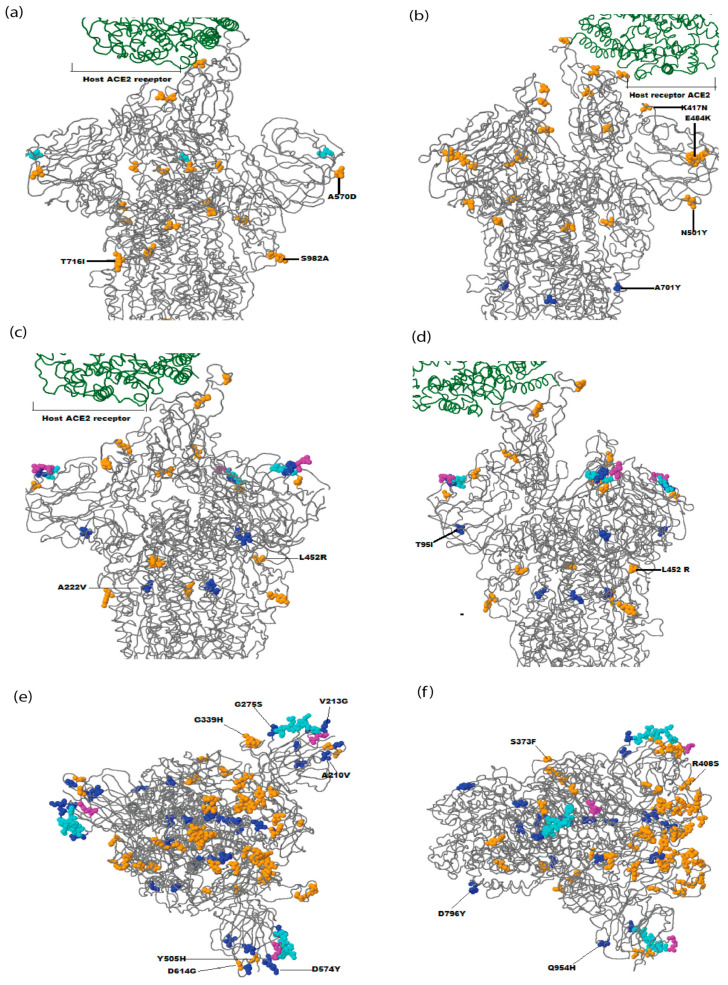
SARS-CoV-2 spike protein with characteristic substitutions in dominant lineages/variants in Pakistan. The figure was constructed online using the GISAID CoVsurver mutations application (https://gisaid.org/database-features/covsurver-mutations-app/). (**a**) B.1.1.7/Alpha having A570D, T716I, and S982A substitutions, (**b**) B.1.351/Beta having K417N, E484K, N501Y, and A501Y substitutions, (**c**) B.1.617.2/Delta having A222V and L453R substitutions, (**d**) AY.108/Delta having T95I and L452R substitutions, (**e**) BA.2.75/Omicron having A210V, V213G, G275S, G339H, Y505H, D574Y, and D614G substitutions, and (**f**) BA.5.2/Omicron having S373F, R408S, D796Y, and Q954H substitutions.

In Pakistan, five waves of the SARS-CoV-2 infection were observed in the past two years. The sequence data reveal six lineages/WHO variants (B.1.1.7/Alpha, B.1.351/Beta, B.1.617.2/Delta, AY.108/Delta, BA.2.75/Omicron, and BA.5/Omicron) were dominant during March 2020–March 2023. The current study also represents the genetic diversity of these lineages/variants. After comparing these sequences with the Wuhan reference sequence, it was found that B.1.1.7/Alpha has 10 amino acid mutations with 99.4% identity, and its prevalence was higher (23.3%). The other variants also show the no. of mutation and % identity, and these include B.1.351/Beta (10, 99.4%), B.1.617.2/Delta (13, 99.1%), AY.108/Delta (11, 99.3%), BA.2.75/Omicron (33, 97.4%), and BA.5.2/Omicron (25, 98.3%) as shown in Table 5 and Figure 4. It was also found that most of the substitutions were found in the N-terminal domain and receptor-binding domain of the gene. The details of these mutations concerning the gene region are presented in Table 5 and Figure 5. The SARS-CoV-2 spike protein structures of these dominant lineages with characteristic mutations are also shown in Figure 6.

## 4. Discussion

The aim of this study was to assess the diversity of SARS-CoV-2 PANGOLIAN lineages and GISAID clades in Pakistan. We sought to understand the evolution of SARS-CoV-2 in the country by identifying amino acid substitutions in the spike protein. Additionally, we analyzed the phylogenetic relationship of Pakistan’s SARS-CoV-2 spike protein sequences with global sequences. A total of 1496 SARS-CoV-2 sequences have been uploaded to the NCBI database since the first sequence was collected in Pakistan in March 2020. Notably, the Sindh province reported the highest number of sequences (*n* = 846), which is attributed to their more efficient disease-reporting surveillance system and genome sequencing capabilities. Our analysis revealed the presence of B4 and B lineages in the first three sequences from Pakistan, and we investigated the ongoing evolution of these lineages by identifying amino-acid-changing substitutions in the spike protein. These findings provide valuable insights into the genetic diversity and evolution of SARS-CoV-2 in Pakistan, which can aid in developing effective disease control interventions [4]. The B4 lineage was the first reported lineage of Iran, having a significant role in the COVID-19 pandemic [5].

The abundance of various SARS-CoV-2 variants in Pakistan has been a subject of increasing concern as the pandemic has progressed. Early in the pandemic, the original wild-type variant dominated, but subsequently, several variants of concern (VOCs) and variants of interest (VOIs) emerged [https://gisaid.org/hcov-19-variants-dashboard/ (accessed on 28 May 2023)]. VOCs such as the Alpha, Beta, Gamma, and Delta variants have displayed increased transmissibility and have been associated with a higher risk of severe disease and mortality. These variants have often outcompeted their predecessors, leading to significant shifts in the viral landscape. Therefore, it is crucial to assess their prevalence and monitor their impact on mortality rates to guide public health strategies in Pakistan. Investigations into the mortality rates associated with different SARS-CoV-2 variants have revealed intriguing patterns. Studies have consistently shown that certain variants, such as the Alpha and Delta variants, have been linked to increased mortality compared to earlier variants [https://outbreak.info/location-reports?loc=PAK/ (accessed on 28 May 2023)]. This finding suggests that the genetic changes in these VOCs might contribute to higher virulence or evasion of the host immune response. Moreover, new variants have occasionally posed challenges regarding treatment efficacy, as certain variants have exhibited reduced susceptibility to therapeutic interventions, potentially impacting patient outcomes. However, it is essential to note that various factors, including healthcare capacity, population demographics, and vaccination rates, influence mortality rates. It is essential to interpret the observed associations between variant abundance and mortality rates within the broader context of the pandemic.

This study investigated the diversity of PANGOLIN lineages and GISAID clades in Pakistan and identified six dominant lineages/variants up to March 2023. We found a total of 96 PANGOLIN lineages circulating in Pakistan. The dominant lineages/variants were B.1.1.7/Alpha, B.1.351/Beta, B.1.617.2/Delta, AY.108/Delta, BA.2.75/Omicron, and BA.5.2/Omicron. The B.1.1.7 lineage (Alpha variant), which emerged in England in September 2020, was first reported in Pakistan in December 2020 and became the dominant lineage in the country. The submission of a higher number of sequences (*n* = 108) in April 2021 further supported its dominance. Previous studies have also reported its higher transmission and virulence than other variants [6,7,8]. Our analysis revealed that this lineage had more substitutions, possibly contributing to its higher transmission and mortality rates. The increased number of B.1.1.7 sequences could be attributed to its higher reproduction rate, as observed in another study in England [6,7,8].

The B.1.351 lineage, also known as the Beta variant, was initially detected in England in early 2020 but is predominantly known as the South African variant. In Pakistan, this lineage was first identified in May 2021, likely due to the travel of overseas Pakistanis from regions where the variant was prevalent. Previous research has indicated that this lineage (B.1.351) is more transmissible than the B.1.1.7/Alpha variant [9,10,11].

The emergence of different lineages and variants of SARS-CoV-2 is a matter of global concern. One of the major lineages reported in Pakistan is the B.1.617.2 lineage/Delta variant, first reported in India. This lineage is highly contagious and virulent in different studies, making it a serious public health concern. Similarly, the AY.108 lineage of the Delta variant has been reported in a higher frequency of sequences (*n* = 76) from Pakistan, highlighting the need to monitor and track the prevalence of this variant. Omicron variants have also been dominant in Pakistan, including BA.2.75 variant, which was first reported in India at the end of 2021. Another major lineage (BA.5.2) of the Omicron variant was found in high frequency (40%) in the United States and was first detected in the middle of 2020.

Interestingly, the present study revealed that B.1.1.7/Alpha, B.1.351/Beta, B.1.617.2/Delta, AY.108/Delta, BA.2.75/Omicron, and BA.5.2/Omicron were the dominant lineages of Pakistan till March 2023. The B.1.1.7/Alpha lineage, first reported in England in September 2020, was the dominant lineage in Pakistan in the initial months. However, previous studies reported the B.1.351/Beta lineage is more transmissible than the B.1.1.7/Alpha variant. Therefore, the emergence of the B.1.351/Beta lineage in Pakistan in May 2021 is a matter of concern as it has been reported to be highly transmissible.

It is worth noting that SARS-CoV-2 has undergone various amino-acid-changing substitutions, particularly in the spike protein, which has a significant role in viral entry into host cells. The Global Initiative on Sharing All Influenza Data (GISAID) database indicates that the S protein has undergone over 1229 amino acid substitutions. Identifying and tracking these amino acid substitutions in different lineages and variants are crucial for understanding their potential impact on viral transmission, virulence, and vaccine efficacy. Therefore, continuous surveillance and monitoring of SARS-CoV-2 lineages and variants are necessary to develop effective public health strategies to combat the pandemic. [12]. The current study investigated the substitutions in the spike protein gene of SARS-CoV-2 in Pakistan to understand the evolution of the virus in the country. The presence of substitutions was analyzed in all reported lineages of Pakistan, including the dominant lineages/variants. Substitutions in all regions of the spike protein of these dominant lineages/variants were also analyzed.

In the B.1.1.7/Alpha lineage, three substitutions (H69del, V70del, and Y144del) were observed in the N-terminal domain of the spike protein. The substitutions N501Y, A570D, and D614G were highly prevalent (>70%) in the RBD region and were considered characteristic of B.1.1.7. Moreover, P681H was identified as the characteristic mutation found in the S1/S2 region.

In the B.1.351/Beta lineage, substitutions D80A, D215G, L242del, A243del, and L244del were observed in the N.T.D. region. In the RBD region, substitutions K417N, E484K, N501Y, and D614G were highly prevalent (>80%) and were considered characteristic of this lineage. The study analyzed two lineages (B.1.617.2 and AY.108) of the Delta variant, and eight substitutions were found in the NTD region of B.1.617.2, whereas six substitutions were found in AY.108. In the RBD region of both lineages, three substitutions were found. L452R and T478K were new substitutions in the Delta variant, and due to their high prevalence, they were considered the characteristic substitutions of these variants.

Regarding the Omicron variant, the study analyzed 2 lineages (BA.2.75 and BA.5.2), and 11 substitutions were found in the NTD region of BA.2.75, while only 3 substitutions were found in BA.5.2. In the RBD region, 17 substitutions were detected, and G446S and N460K were found only in BA.2.75 and, hence, were considered the characteristic substitutions of this lineage of the Omicron variant.

The present study provides insights into the substitutions present in the spike protein gene of SARS-CoV-2 in Pakistan and the characteristic substitutions of different lineages/variants. These findings can aid in the development of effective vaccines and treatments against COVID-19. Based on previous studies, the spike protein N-terminal domain (NTD) of SARS-CoV-2 contains epitopes that are targeted by neutralizing antibodies generated by the host’s adaptive immune system [13,14]. Moreover, the NTD has been implicated in the interaction between the spike protein and glycosylated components of the host cell surface, which plays a crucial role in host cell adherence. Consequently, it is crucial to monitor the genetic diversity of the NTD to track the emergence of new viral variants. Our investigation indicates that specific loop components of the NTD, which have evolved differently among various SARS-CoV-2 clades, exhibit significant mutation rates. This finding underscores the importance of continuous surveillance and monitoring of the NTD of the spike protein to better understand the virus’s evolutionary dynamics and its potential impact on disease transmission and severity [13,14].

The substitutions occurring in the receptor-binding domain (RBD) of the spike (S) protein, play a critical role in the susceptibility of SARS-CoV-2 variants to both antibodies and infectiousness. Previous research has identified seven dominant RBD substitutions (G339D, N440K, L452R, S477N, T478K, E484K, and N501Y) that have been shown to enhance the positively charged residue interactions with ACE2 [15]. Of these substitutions, the D614G mutation has been the most prevalent (79%) and has been associated with increased viral transmission. It has been hypothesized that the substitution of aspartic acid with glycine at residue 614 enhances the virus’s infectivity [16]. These findings suggest that monitoring the emergence and prevalence of RBD substitutions is essential for understanding the evolution of SARS-CoV-2 variants and their potential impact on disease transmission and severity. Further investigation is needed to fully elucidate the functional consequences of these substitutions and their impact on the efficacy of existing therapeutics and vaccine strategies.

After analyzing the results of this study, there are several limitations that need to be considered. Firstly, our study only focused on analyzing the spike protein of SARS-CoV-2 in Pakistan, and thus the findings may not be generalizable to other countries or regions. Moreover, due to the rapid evolution of the virus, the analysis of sequenced samples may not capture all of the mutations that have occurred since the time of sampling. Furthermore, while our study provides insights into the evolutionary trend of SARS-CoV-2 in Pakistan, it does not provide information on the clinical or epidemiological implications of the observed mutations. Finally, the structural analysis of the spike protein was limited to substitutions in the dominant lineages, and future studies could explore other potential substitutions that may affect viral behavior. Therefore, caution should be exercised when interpreting the results of this study, and further research is required to fully understand the implications of the observed mutations.

In conclusion, the present study sheds light on the rapid evolution of SARS-CoV-2 in Pakistan by analyzing 96 lineages representing the country’s diverse clades. Our findings reveal numerous variations and amino acid substitutions over time, indicating a highly dynamic viral landscape. Our comparative residue analysis provides accurate insights into the dominant lineages and their structural changes. These results hold significant implications for understanding the evolutionary trend of SARS-CoV-2 in Pakistan and beyond. By identifying the pattern of substitutions in dominant lineages, this study highlights the importance of monitoring and adapting to the rapidly evolving virus. Ultimately, our research provides valuable information for developing effective strategies to combat the ongoing pandemic.

## Figures and Tables

**Table 1:** Distribution of the GRA Clade of Omicron variant and corresponding lineages of SARS-CoV-2 Detected in Pakistan.

GISAID			PANGOLIN			WHO	Geographical Distribution
Clade	No. of Sequences	Percentage	Lineage	No. of Sequences	Percentage	Variant	Province
GRA	474	34.27	B.1.1.529	3	0.22	Omicron	Sindh
			BA.1	22	1.59	Omicron	Sindh
			BA.1.1	44	3.18	Omicron	Sindh
			BA.1.1.1	2	0.14	Omicron	Sindh
			BA.1.1.18	1	0.07	Omicron	Sindh
			BA.1.15.1	1	0.07	Omicron	Sindh
			BA.1.17.2	1	0.07	Omicron	Sindh
			BA.1.18	1	0.07	Omicron	Sindh
			BA.1.21	2	0.14	Omicron	Sindh
			BA.2	61	4.41	Omicron	Sindh
			BA.2.10.1	1	0.07	Omicron	Sindh
			BA.2.12.1	3	0.22	Omicron	Sindh
			BA.2.14	1	0.07	Omicron	Sindh
			BA.2.15	1	0.07	Omicron	Sindh
			BA.2.3	1	0.07	Omicron	Sindh
			BA.2.36.1	2	0.14	Omicron	Sindh
			BA.2.5	1	0.07	Omicron	Sindh
			BA.2.75.1	1	0.07	Omicron	Sindh
			BA.2.75.2	1	0.07	Omicron	Sindh
			BA.4	7	0.51	Omicron	Sindh
			BA.4.1	8	0.58	Omicron	Sindh
			BA.5.1	1	0.07	Omicron	Sindh
			BA.5.2	205	14.82	Omicron	Sindh
			BA.5.2.1	37	2.68	Omicron	Sindh
			BA.5.2.16	8	0.58	Omicron	Sindh
			BA.5.2.18	1	0.07	Omicron	Sindh
			BA.5.2.20	1	0.07	Omicron	Sindh
			BA.5.2.28	1	0.07	Omicron	Sindh
			BA.5.2.3	1	0.07	Omicron	Sindh
			BA.5.2.56	2	0.14	Omicron	Sindh
			BA.5.2.6	1	0.07	Omicron	Sindh
			BA.5.2.9	1	0.07	Omicron	Sindh
			BA.5.3	1	0.07	Omicron	Sindh
			BA.5.3.1	1	0.07	Omicron	Sindh
			BA.5.5	2	0.14	Omicron	Sindh
			BA.5.6	2	0.14	Omicron	Sindh
			BQ.1	3	0.22	Omicron	Sindh
			BQ.1.1	3	0.22	Omicron	Sindh
			BQ.1.13	1	0.07	Omicron	Sindh
			XBB.1	18	1.3	Omicron	Punjab, Sindh
			XBB.1.11.1	1	0.07	Omicron	Sindh
			XBB.1.12	1	0.07	Omicron	Sindh
			XBB.1.5	4	0.29	Omicron	Punjab, Sindh
			XBB.1.5.9	1	0.07	Omicron	Sindh
			XBB.1.9	1	0.07	Omicron	Sindh
			XBB.1.9.1	5	0.36	Omicron	Punjab, Sindh
			XBB.1.9.2	3	0.22	Omicron	Punjab, Sindh
			XBB.2	1	0.07	Omicron	Sindh
			XBB.2.1	1	0.07	Omicron	Sindh
			XBB.2.4	1	0.07	Omicron	Sindh
Total	474						

**Table 2:** Distribution of L, O, S, G, GH, GK, GR, GRY clades and corresponding lineages of SARS-CoV-2 detected in Pakistan along with WHO variant names.

GISAID			PANGOLIN			WHO	Geographical Distribution
Clade	No. of Sequences	Percentage	Lineage	No. of Sequences	Percentage	Variant	Province
L	18	1.3	B	14	1.01	-	KPK, Sindh, and Punjab
			B.4	4	0.29	-	Sindh, Punjab, and Gilgit
O	15	1.08	B.1.260	1	0.07	-	Sindh
			B.6	13	0.94	-	Punjab, Sindh, KPK, and AJK
			B.6.6	1	0.07	-	Sindh
S	6	0.43	A	6	0.43	-	Punjab and Sindh
G	73	5.27	B.1.617.2	73	5.28	Delta	Punjab and Sindh
GH	225	16.26	B.1	127	9.18	Beta	KPK, Sindh, and Punjab
			B.1.351	36	2.6	Beta	Punjab and Sindh
			B.1.36	24	1.74	-	Punjab and Sindh
			B.1.36.24	3	0.22	-	Punjab
			B.1.36.31	10	0.72	-	Punjab and Sindh
			B.1.36.34	2	0.14	-	Punjab
			B.1.471	21	1.52	-	Punjab and Sindh
			B.1.525	2	0.14	-	Sindh
GK	114	8.24	AY.106	1	0.07	Delta	Sindh
			AY.108	85	6.15	Delta	Punjab, Sindh, and KPK
			AY.126	6	0.43	Delta	Punjab and Sindh
			AY.127	12	0.87	Delta	Sindh
			AY.46	1	0.07	Delta	Sindh
			AY.46.2	1	0.07	Delta	Sindh
			AY.55	6	0.43	Delta	Punjab and Sindh
			AY.65	2	0.14	Delta	Punjab and Sindh
GR	48	3.47	AE.4	1	0.07	-	Sindh
			B.1.1	21	1.52	-	Punjab and Sindh
			B.1.1.1	21	1.52	-	Punjab, KPK, and AJK
			B.1.1.413	1	0.07	-	Sindh
			C.23	4	0.29	-	Punjab
GRY	328	23.71	B.1.1.7	326	23.57	Alpha	Punjab and Sindh
			BQ.1.5	1	0.07	Alpha	Sindh
			B.1.1.75	1	0.07	Alpha	Punjab
Total	827						

**Table 3:** Distribution of Unclassified Group (NA) SARS-CoV-2 Sequences and Lineages Detected in Pakistan.

GISAID			PANGOLIN			WHO	Geographical Distribution
Clade	No. of Sequences	Percentage	Lineage	No. of Sequences	Percentage	Variant	Province
NA	195	14.09	Q.4	1	0.07	-	Punjab
			BE.3	3	0.22	-	Sindh
			BF.5	2	0.14	-	Sindh
			BN.1	1	0.07	-	Sindh
			BN.1.3	2	0.14	-	Sindh
			BN.1.3.4	1	0.07	-	Sindh
			BN.1.4	1	0.07	-	Sindh
			BV.2	6	0.43	-	Sindh
			BY.1	1	0.07	-	Sindh
			CK.1	44	3.18	-	Sindh
			CK.1.2	1	0.07	-	Sindh
			CK.2	13	0.94	-	Sindh
			CK.2.1	7	0.51	-	Sindh
			CR.1	2	0.14	-	Sindh
			CT.1	1	0.07	-	Sindh
			unclassifiable	109	7.88	-	Punjab, Sindh, and KPK
Total	195						

**Table 4:** Amino acid substitutions with the frequency of occurrence and percentage detected in Pakistan sequences.

Substitutions	Frequency of Occurrence (*N* = 44)	Percentage
D614G	35	79.54
T478K	26	59.09
G142D	25	56.81
N501Y and P681H	22	50
S477N and E484A	21	47.72
V211G, G339D, K417N, N440K, C498R, Y505H, H655Y, N679K, D796Y, C954H, and N969K	20	45.45
S373P and S375F	20	44.45
T19I, L24S, T376A, and D405N	18	40.90
S371F, R408S, and F486V	13	29.54
V68I	12	27.27
N764K	9	20.45
D950N	6	13.63
T19R, E156G, N460K, C493R, and P681R	5	11.36
R346K, K444R, L452C, E484K, N658S, A570T, A570D, T572I, D574V, N658Y, S673T, A701V, S704L, T716I, K814R, A845S, A846V, I850L, D950H, S982A, A1020S, T1117I, D1118H, E1182C, and E1202C	1	2.27

**Table 5:** Mutational diversity present in the spike protein of B.1.1.7/Alpha, B.1.351/Beta, B.1.617.2/Delta, AY.108/Delta, BA.2.75/Omicron, and BA.5.2/Omicron lineage/variant in Pakistan.

S Gene Region	B.1.1.7/ Alpha	B.1.351/ Beta	B.1.617.2/ Delta	AY.108/ Delta	BA.2.75/ Omicron	BA.5.2/ Omicron
**NTD**	H69del	D80A	T19R	T19R	T19I	T19I
	V70del	D215G	-	T95I	L24del	-
	Y144del	L242del	-	-	A27S	-
	-	A243del	G142D	G142D	G142D	G142D
	-	L244del	E156G	E156G	K147E	-
	-	-	F157del	F157del	W152R	-
	-	-	R158del	R158del	F157L	-
	-	-	V159X	-	I210V	-
	-	-	Y160X	-	V213G	-
	-	-	A222V	-	G257S	-
	-	-	-	-	G339H	G339D
**RBD**	-	-	-	-	S371F	S371F
	-	-	-	-	S373P	S373P
	-	-	-	-	S375F	S375F
	-	-	-	-	T376A	T376A
	-	-	-	-	D405N	D405N
	-	-	-	-	R408S	R408S
	-	K417N	-	-	K417N	K417N
	-	-	-	-	N440K	N440K
	-	-	L452R	L452R	S477N	S477N
	-	-	T478K	T478K	T478K	T478K
	-	E484K	-	-	E484A	E484A
	-	-	-	-	Q498R	Q498R
	N501Y	N501Y	-	-	N501Y	N501Y
	A570D	-	-	-	Y505H	Y505H
	D614G	D614G	D614G	D614G	D614G	D614G
**SD2**	-	-	-	-	H655Y	H655Y
	-	-	-	-	N679K	N679K
**S1/S2**	P681H	-	P681H	P681H	P681H	P681H
**UH**	T716I	A701V	-	-	N764K	N764K
**FP.**	-	-	-	-	D796Y	D796Y
**HR1**	S982A	-	D950N	D950N	Q954H	Q954H
	-	-	-	-	N969K	N969K
**CD**	D1118H	-	-	-	-	-
**No. of Substitutions**	**10**	**10**	**13**	**11**	**33**	**25**
**% AA Identity**	**99.4%**	**99.4%**	**99.1%**	**99.3%**	**97.4%**	**98.3%**

## Data Availability

The datasets analyzed during the current study were retrieved from and are available in the NCBI repository [https://www.ncbi.nlm.nih.gov/sars-cov-2/ (accessed on 5 March 2023)]. The accession numbers and details of these SARS-CoV-2 sequences are shown in the Supplementary Materials.

## Supplementary Materials

The following supporting information can be downloaded at: https://www.mdpi.com/article/10.3390/v15071450/s1; Table S1: A Catalogue of SARS-CoV-2 Sequences Originating from Pakistan.
